# Anti-BCMA CAR-T cells attenuate microglial activation in progressive multiple sclerosis: indicating a plasma cell-microglia crosstalk

**DOI:** 10.1038/s41392-025-02510-6

**Published:** 2025-12-20

**Authors:** Fatme S. Ismail, Hans-Peter Hartung, Nico Melzer

**Affiliations:** 1 Department of Neurology, Knappschaft Kliniken Vest, Academic Teaching Hospital of the Ruhr University Bochum, Recklinghausen, Germany; 2 Department of Neurology, Medical Faculty and University Hospital, Heinrich-Heine University of Düsseldorf, Düsseldorf, Germany; 3 Brain and Mind Center, University of Sydney, Sydney, NSW Australia; 4 Department of Neurology, Palacky University Olomouc, Olomouc, Czech Republic

**Keywords:** Neurological disorders, Neuroimmunology

A recent publication by Qin et al. in Cell reported on the first-in-human application of anti-BCMA chimeric antigen receptor (CAR)-T cell therapy in patients with treatment-refractory progressive multiple sclerosis (MS) with a good tolerability and efficacy.^1^

CAR-T cells rank among the breakthrough therapeutic approaches, transforming the field of cancer and autoimmune conditions including neuroimmunological disorders. B-cell maturation antigen (BCMA) and CD19 antigen are B-cell target antigens frequently used in CAR constructs for autoimmune diseases.^1,2^ In addition to the B-cell depletion in the peripheral blood, CAR-T cells allow deep depletion of tissue-resident B cells e.g., in lymphoid tissues and hard-to-reach compartments such as the central nervous system (CNS), an effect that has not been observed with antibody-based B-cell depletion therapies. However, little is known about direct and indirect effects of CAR-T cells on microglia, even though CNS-resident microglial cells are involved as one of the key drivers and maintainers of the compartmentalized neuroinflammation and neurodegeneration during MS progression. This is confirmed by emerging evidence that smoldering neurodegenerative processes are hallmarked by chronic microglia-mediated neuroinflammation with progressive axonal injury, resulting in disability progression independent of relapse activity (PIRA) and brain atrophy.^1^ To date, none of the available therapies for MS are fully effective at preventing disease progression and PIRA.

**Fig. 1:**
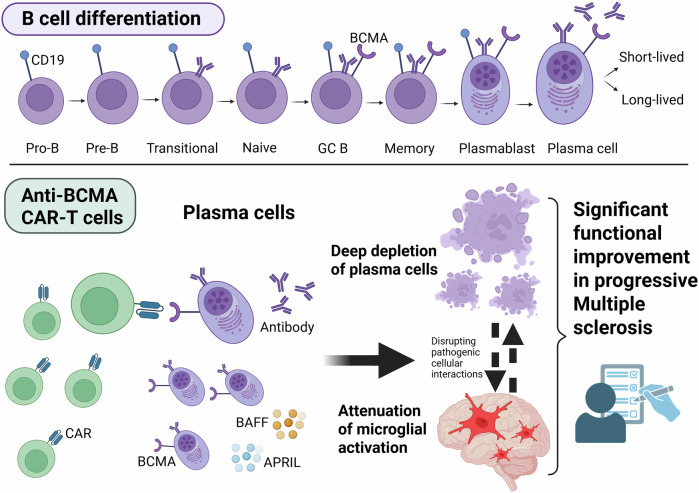
During B cell differentiation, B-cell maturation antigen (BCMA) is expressed on late-stage B cells (after differentiation to immunoglobulin-secreting cells), short-lived plasmablasts and long-lived plasma cells, as recently shown with a BCMA reporter mouse model. However, differences in BCMA expression between mouse and human immune cells have been found and should be considered. BCMA is necessary for the survival of long-lived plasma cells, production of antibodies, class switch of immunoglobulins. A proliferation-inducing ligand (APRIL) and B-cell activating factor (BAFF) are physiological ligands for BCMA. CD19 and BCMA are the most frequently used B-cell target antigens in CAR constructs for autoimmune diseases. Anti-BCMA CAR-T cell treatment in progressive Multiple sclerosis led to deep depletion of BCMA-expressing B cells/plasma cells in compartments of the central nervous system and attenuated microglial activation, resulting in significant functional improvement during a 9-month follow-up. The intriguing effect of anti-BCMA CAR-T cells on microglia might indicate a plasma cell-microglia crosstalk. CAR chimeric antigenreceptor, CD19 cluster of differentiation 19, GC B germinal center B cells. Figure created in BioRender. Ismail, F. (2025) https://BioRender.com/z2pozz2

The emerging study by Qin et al. showed, for the first time, significant functional improvement during a 9-month follow-up in five patients with primary/secondary progressive MS following anti-BCMA CAR-T cell treatment.^1^ Further findings of the study include: reduction of cerebrospinal fluid (CSF) immunoglobulin levels and kappa-free light-chain; CNS penetration of anti-BCMA CAR-T cells which delayed expand and longer persist in the CSF compared to peripheral blood and bone marrow; B cell/plasma cell depletion in CNS compartments; particularly noteworthy, attenuation of microglial activation, providing insights into CAR-T cell effects on microglia-mediated CNS neuroinflammation (Fig. 1). Before starting treatment, the authors have analyzed inflammatory pathways of various immune cell types in the CSF and have identified myeloid cells as the leading clusters driving neuroinflammation. After further re-clustering into macrophage-like and microglia-like cells, B cells and microglia-like cells had the strongest interactions in the enrolled MS patients. These findings support that B cell-activated microglia-like cells could play a pivotal role in driving and maintaining neuroinflammation in progressive MS. Effective depletion of B cells with anti-BCMA CAR-T cells resulted in a significant downregulation of the inflammation-associated changes in microglia-like cells and mitigated the intensity of interactions between various immune cells in the CSF, accompanied by decreased levels of pro-inflammatory cytokines. In addition, positron emission tomography (PET) targeting a second-generation translocator protein TSPO (TSPO-PET), an innovative marker for in vivo imaging of microglial activity states, confirmed a significant attenuation of microglial activation in both primary and secondary progressive MS patients following anti-BCMA CAR-T cell therapy. Underlying pathomechanisms could include the tumor necrosis factor alpha (TNF-α)/nuclear factor κB (NF-κB) pathway through disruption of the pathogenic B cell-to-microglia signaling axis. Interestingly, BCMA is expressed not only on B cells/plasma cells, but also found in microglia and macrophages in the CNS. In experimental autoimmune encephalitis (EAE) model, stimulation through BAFF (B-cell activating factor) and APRIL (a proliferation-inducing ligand) increased secretion of the pro-inflammatory cytokine interleukin-6 in BCMA-expressing macrophages, providing evidence that the pro-inflammatory effect of BAFF is accomplished specifically through BCMA on macrophages.^3^ In line with another study, elimination of BAFF in B cell-deficient mice significantly reduced EAE severity, suggesting that BAFF contributes to autoimmunity not only through effects on B cells but also through B cell-independent pathways. These findings raise the question of whether anti-BCMA CAR-T cells could have, aside from indirect effects, additional direct effects on BCMA-expressing microglia and macrophages involved in MS pathogenesis. Another noteworthy point is that BCMA RNA expression is detected on neurons and astrocytes in the caudate nuclei of normal human brains according to public transcriptomic datasets. In patients with hematological malignancies, progressive movement disorder with features of Parkinsonism has been described multiple weeks to months after anti-BCMA CAR-T cell treatment. Post-mortem investigations showed BCMA expression on neurons and astrocytes in the basal ganglia of a patient, indicating a rare, on-target off-tumor toxicity effect of the anti-BCMA CAR-T cell therapy.^4^ However, further investigations are needed to confirm the BCMA expression in the CNS and the possible effects of anti-BCMA CAR-T cells.

The significant functional neurological improvement in five patients treated with anti-BCMA CAR-T cells (follow-up of 9 months) was not obtained for treatment of two patients with progressive MS following anti-CD19 CAR-T cells, with the longest follow-up of 100 days.^1,2^ It was discussed that CD19-targeting CAR-T cells can penetrate the CNS, but are hardly capable of eliminating long-lived plasma cells. These findings, especially the clinical effects, should be interpreted with caution due to the low number of patients and the observation period. Also, the lymphodepleting chemotherapy with fludarabine and cyclophosphamide administered before the transfer of anti-BCMA CAR-T cells could exert short-term therapeutic effects, but not explaining the specific effects related to CAR-T cells such as deep depletion and CNS penetration.

Moreover, CAR-T cells targeting CD19, GD2 or B7H3 for CNS and non-CNS cancers induced white matter microglial reactivity with persistent neuroinflammation, dysfunction of oligodendroglial cells and restricted hippocampal neurogenesis in several mouse models, resulting in long-term cognitive impairment.^5^ Additionally, microglial and oligodendroglial reactivity were detected also during post-mortem examination of the frontal cortex and subcortical white matter of patients treated with anti-GD2 CAR-T cell therapy for brainstem tumors. In mouse models, microglial depletion improved the cognitive impairments following CAR-T cell therapy targeting CD19 or GD2.^5^ All these findings should be considered in the context of the disease being treated (cancer vs autoimmunity) and the type of CAR-T cell therapy administered (especially target antigens). Also, microglial features and functions vary depending on the disease pathogenesis. Finally, CAR-T cell constructs could exert different effects on glial cells depending on disease being treated and the targets of the therapy (BCMA, CD19 or other antigens).

In conclusion, anti-BCMA CAR-T cells could have the potential of a single one-off treatment with long-term effect on chronic B-cell-/microglia-driven neuroinflammation and accompanied neurodegeneration, representing a real game-changer in the future management of MS. Several ongoing international trials are evaluating CAR-T cells for MS treatment. Recruiting larger patient cohorts and collecting long-term data (incl. monitoring of microglial activation states e.g., using TSPO-PET), as well as conducting further experimental studies, can shed light on the true impact of CAR-T cells on MS pathogenesis and clinical outcome.
